# Immersive Virtual Reality Exergames to Promote the Well-being of Community-Dwelling Older Adults: Protocol for a Mixed Methods Pilot Study

**DOI:** 10.2196/32955

**Published:** 2022-06-13

**Authors:** Samira Mehrabi, John E Muñoz, Aysha Basharat, Jennifer Boger, Shi Cao, Michael Barnett-Cowan, Laura E Middleton

**Affiliations:** 1 Department of Kinesiology University of Waterloo Waterloo, ON Canada; 2 Department of Systems Design Engineering University of Waterloo Waterloo, ON Canada; 3 Research Institute for Aging Waterloo, ON Canada

**Keywords:** virtual reality, exergames, community-dwelling older adults, pilot protocol, feasibility, well-being, physical activity, cognition, perception, mood, COVID-19

## Abstract

**Background:**

Despite the proven benefits of exercise in older adults, challenges such as access and motivation can deter their engagement. Interactive virtual reality (VR) games combined with exercise (exergames) are a plausible strategy to encourage physical activity among this population. However, there has been little research on the feasibility, acceptability, and potential benefits of deploying at-home VR exergames among community-dwelling older adults.

**Objective:**

The objectives of this study are to estimate the feasibility, usability, and acceptability of a co-designed VR exergame in community-dwelling older adults; examine intervention feasibility and assessment protocols for a future large-scale trial; and provide pilot data on outcomes of interest (physical activity, exercise self-efficacy, mood, cognition, perception, and gameplay metrics).

**Methods:**

The study will be a remote, 6-week intervention comprising an experimental and a control group. A sample of at least 12 community-dwelling older adults (with no or mild cognitive impairment) will be recruited for each group. Both groups will follow the same study procedures and assessment methods. However, the experimental group will engage with a co-designed VR exergame (*Seas The Day*) thrice weekly for approximately 20 minutes using the Oculus Quest 2 (Facebook Reality Labs) VR headset. The control group will read (instead of playing *Seas The Day*) thrice weekly for approximately 20 minutes over the 6-week period. A mixed methods evaluation will be used. Changes in physical activity, exercise self-efficacy, mood, cognition, and perception will be compared before and after acute data as well as before and after the 6 weeks between the experimental (exergaming) and control (reading) groups. Qualitative data from postintervention focus groups or interviews and informal notes and reports from all participants will be analyzed to assess the feasibility of the study protocol. Qualitative data from the experimental group will also be analyzed to assess the feasibility, usability, and acceptability of at-home VR exergames and explore perceived facilitators of and barriers to uptaking VR systems among community-dwelling older adults.

**Results:**

The screening and recruitment process for the experimental group started in May 2021, and the data collection process will be completed by September 2021. The timeline of the recruitment process for the control group is September 2021 to December 2021. We anticipate an estimated adherence rate of ≥80%. Challenges associated with VR technology and the complexity of remote assessments are expected.

**Conclusions:**

This pilot study will provide important information on the feasibility, acceptability, and usability of a custom-made VR exergaming intervention to promote older adults’ well-being. Findings from this study will be useful to inform the methodology, design, study procedures, and assessment protocol for future large-scale trials of VR exergames with older adults as well as deepen the understanding of remote deployment and at-home use of VR for exercise in older adults.

**International Registered Report Identifier (IRRID):**

DERR1-10.2196/32955

## Introduction

### Background

Population aging is a global phenomenon. With the increase in global life expectancy (average of 73.4 years in 2019) and all baby boomers reaching >65 years of age in 2031 [1], the proportion of older adults (aged ≥60 years) is estimated to nearly double (from 12% to 22%) and increase to upwards of 2 billion individuals by 2050 worldwide [2]. The number of *oldest-old* individuals (aged ≥80 years) is increasing even faster and is expected to triple by 2050 [3]. With such a drastic shift in global population demographics, the incidence of chronic conditions and cognitive impairment is also expected to increase exponentially, leading to higher rates of disability and dependency worldwide [4-6]. In addition to decreased functional ability, cognitive changes, specifically in executive function, pose significant health and social challenges to older adults’ everyday lives and independence [7-9]. This warrants research focused on strategies to foster healthy aging and reduce the impact of age-related changes in older adults’ functional and cognitive abilities.

Physical activity is an established predictor of healthy aging and is associated with numerous physical and psychological health benefits in older adults. Incorporating physical activity into daily life can improve older adults’ motor function, mood, cognition, quality of life, and independence [10-12]. An extensive body of literature has highlighted the positive effects of physical activity on older adults’ executive functions, including processing speed, attention, inhibition, and working memory [13,14]. In particular, both a single bout of aerobic exercise and a period of aerobic exercise training have been shown to promote brain health and executive function in the older population [12,15-17]. Physical activity has also been recognized as a valuable part of preventative and therapeutic strategies for older adults living with mild cognitive impairment and people living with dementia [12,18-21]. Despite strong evidence supporting the broad physical and mental benefits of physical activity, older adults tend to be the least physically active age group, and few older adults are sufficiently active to meet the physical activity guidelines [22]. Lack of motivation and enjoyment, limited access to exercise programs or equipment, and physical limitations are some of the main reasons cited for physical inactivity among older adults [23]. For those living with cognitive impairment, physical activity participation and adherence are particularly restricted because of health challenges associated with the condition as well as poor self-efficacy, apathy, and poor access to exercise opportunities that meet their needs and capabilities [24-26].

### Physical Activity in the Era of the COVID-19 Pandemic

Barriers to physical activity have been exacerbated during the COVID-19 pandemic. Countries have implemented public health measures, including program and facility closures, to contain COVID-19 infection and reduce its health, social, and economic impacts. Older adults are considered a highly vulnerable population to the impact of the COVID-19 outbreak and have faced stricter restrictions than other age groups [27,28]. Staying physically active during the COVID-19 pandemic is more difficult but is particularly important as physical activity is a protective factor against viral infections [29,30]. In addition, engaging in physical activity can be a moderating factor for mental well-being [31-33].

However, with physical distancing measures and the closure of exercise programs and fitness facilities in response to the COVID-19 pandemic, physical activity and exercise opportunities are more restricted for many older adults [34,35]. More optimistically, restrictions and changes in lifestyle as a result of COVID-19 have increased technology adoption among older adults [36-39]. Various opportunities for using novel information and communication technologies (eg, mobile apps, activity trackers, and gamified exercises) have emerged aiming to complement remote exercise programs and potentially benefit older adults’ health and well-being while mitigating social distancing [40,41]. From wearables to applied games, digitally connected technologies are now filling the gap in many in-person activities that cannot be conducted because of the COVID-19 pandemic [42].

When promoting at-home physical activity, exercise video games (also known as exergames) have been shown to be a viable supportive tool to enhance older adults’ well-being during the current pandemic [43-46]. Systematic reviews have shown that exergames have been successfully used to foster improvements in health-related outcomes such as pain management, posture, cognitive functioning, and decreased risk of falls among community-dwelling older adults [47]. Specific physical outcomes such as muscle strength, balance, mobility (including upper and lower limb flexibility), and cardiorespiratory fitness also improved after exposing prefrail and frail older adults to exergaming interventions [48]. Similar results were found in active older adults using exergames with floor-projected features [49]. Although Nintendo Wii and Microsoft Kinect are among the most used interactive systems, the use of immersive virtual reality (VR)—which includes a head-mounted display—is still scarce [50]. Advancements in hardware development, distribution, and accessibility are essential to increase the use of VR applications. The creation of more immersive VR technologies, which are now accessible and commercially available, has allowed for the distribution of VR-based exergames to aid the immersion and interaction of players in virtual environments and potentially create an engaging experience to encourage physical activity participation [51]. The main difference of VR exergames compared with traditional exergames is the use of head-mounted displays to provide a more immersive experience, potentially leading to a more consistent feeling of presence and agency [52]. However, deploying VR exergaming technology for older adults’ at-home exercise, especially during a pandemic, can be complex for various reasons, such as (1) technology access (eg, the cost is still similar to state-of-the-art video gaming consoles) [47], (2) technical complexities (eg, hardware calibration and launching the games as well as content appropriateness), (3) older adults’ technology literacy, and (4) challenges of remote deployment and testing to capture potential physical and mental outcome measures [52]. Moreover, although the multisensory environment of VR exergames can be tailored to the participants’ functional and cognitive abilities [53], research on preferences for VR activities and interactions among older adults and systematic evaluation of VR exergames is limited [54]. This makes the creation and deployment of home-based VR exergames for older adults challenging; and to our knowledge, it has not been done remotely before.

The long-term objective of our research is to develop and assess VR exergames that enable greater access to exercise opportunities for older adults. Therefore, the proposed pilot study aims to determine the feasibility, usability, and acceptability of a 6-week VR exergame intervention and the feasibility of the assessment protocol among community-dwelling older adults. Changes in potential outcomes for a larger-scale trial will also be explored, including physical activity, exercise self-efficacy, mood, cognition, and perception. The pilot gameplay data enable an exploration between game metrics and outcome performance, which will allow for the estimation of whether data from the game can be used for real-time assessments and other such applications.

## Methods

### Study Design

This is a 6-week pilot study to examine the feasibility, acceptability, and usefulness of a co-designed VR exergame intervention among older adults as well as the feasibility of the study procedures and assessment protocol. A control group will be used to account for practice and exposure effects and will follow the same protocol as that described for the experimental group without exposure to the exergame; they will read 3 times a week for 15 to 20 minutes instead of engaging with our exergames. To maintain consistency between the control and experimental conditions, the same questionnaires and surveys will be completed by all the participants in both groups, except for the *perceived enjoyment* and *rate of perceived exertion (RPE)* scales, which will be completed only by the experimental group. Both qualitative and quantitative methods will be used to provide pilot data on potential outcomes of interest. Although this study will evaluate the feasibility of both the study intervention and study control arms, we will not randomize the participants. Participants in the intervention arm will be recruited first, followed by a wave of participants recruited to the control group. The study is based in Waterloo but will be carried out remotely, so participants from across Canada will be eligible.

### Study Sample and Recruitment

A combination of convenience and snowball sampling will be used for the purposes of this study. To be eligible, participants must be aged ≥60 years, living in a community setting in Canada, and able to communicate in English and provide consent on their own behalf. The participants must have access to a computing device (computer or tablet) and a reliable internet connection at their place of residence to complete the web-based assessments and tasks. Participants in both groups must be able to safely participate in light to moderate unsupervised activity without requiring a physician’s approval as assessed using the Get Active Questionnaire (GAQ) [55,56]. Exclusion criteria include (1) diagnosis of moderate or severe dementia or a score <18 on the Montreal Cognitive Assessment (MoCA; version 8.1-Telemed) [57,58]; (2) self-reported severe motion sickness or commonly experienced motion sickness or nausea when driving or sitting in a car, train, or bus; (3) self-reported hearing impairment that may interfere with the participant’s ability to hear and understand the auditory cues in the cognitive and perceptual tasks; (4) uncorrected visual impairment (eg, cataracts, glaucoma, or macular degeneration) that may interfere with the participant’s ability to see and interact with the game and perform the cognitive and perceptual tasks; (5) ear infection in the previous 12 months or a diagnosis of a disease of the middle ear, such as Meniere disease; and (6) any preexisting conditions that would prevent them from engaging in exercise, including having a cardiac pacemaker. The same inclusion and exclusion criteria will be used for both the experimental and control groups to ensure internal validity.

### VR Intervention

#### Software

For the VR exergame intervention in this study, the participants will play a custom-made VR exergame, *Seas The Day*, on an Oculus Quest 2 (Facebook Reality Labs) headset [59,60]. *Seas The Day* was designed using an extensive user-centered and participatory design process with various stakeholders, including older adults, exercise professionals, game designers, engineers in the field of human factors and assistive technologies, and a local VR studio [61]. The VR exergame simulates a remote tropical island with several different activities that are intended to encourage upper-body physical activity. The game is designed to be played seated so that fall risk is minimized. Each exergaming session lasts 15 to 20 minutes.

**Figure 1:**
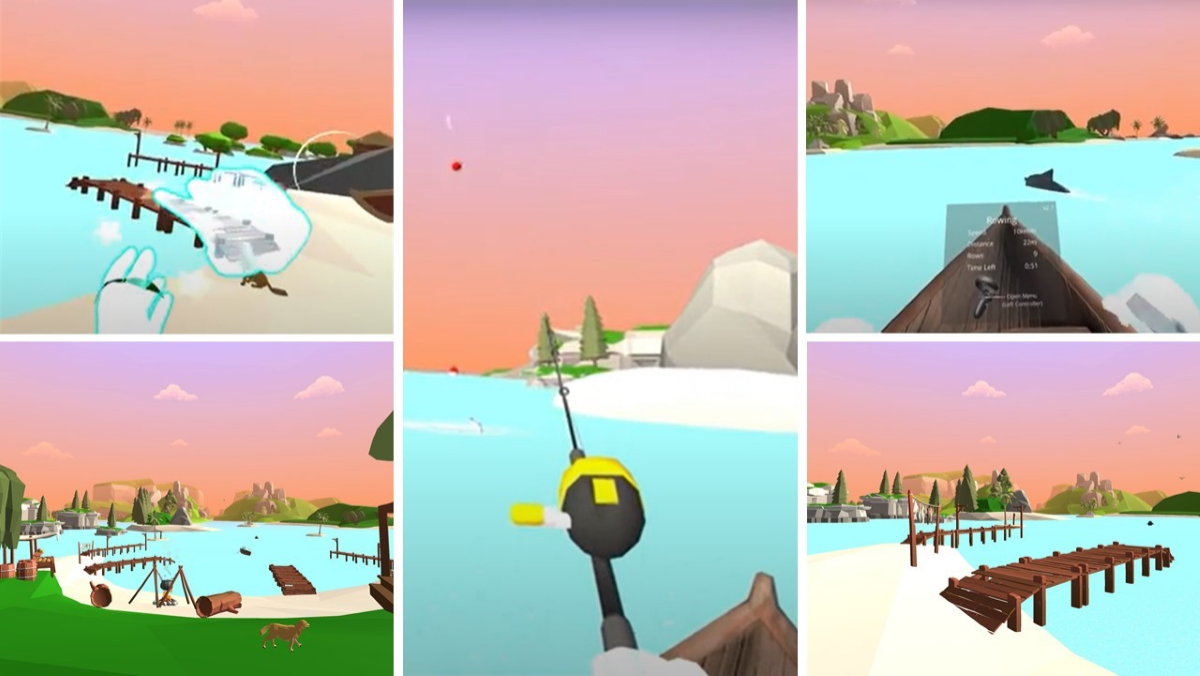
Screenshots of the *Seas The Day* exergame showing the tropical virtual environment and some of the activities.

*Seas The Day* (Figure 1) includes 3 virtual locations with three different activities: (1) a tai chi warm-up, (2) a rowing conditioning exercise, and (3) a fishing cooldown. Recorded audio clips guide the participants on game activities alongside visuo-tactile cues to facilitate interaction. No buttons are needed to interact with the virtual elements. Players are automatically transported from one game stage to the next to prevent interaction errors.

#### Hardware

**Figure 2:**
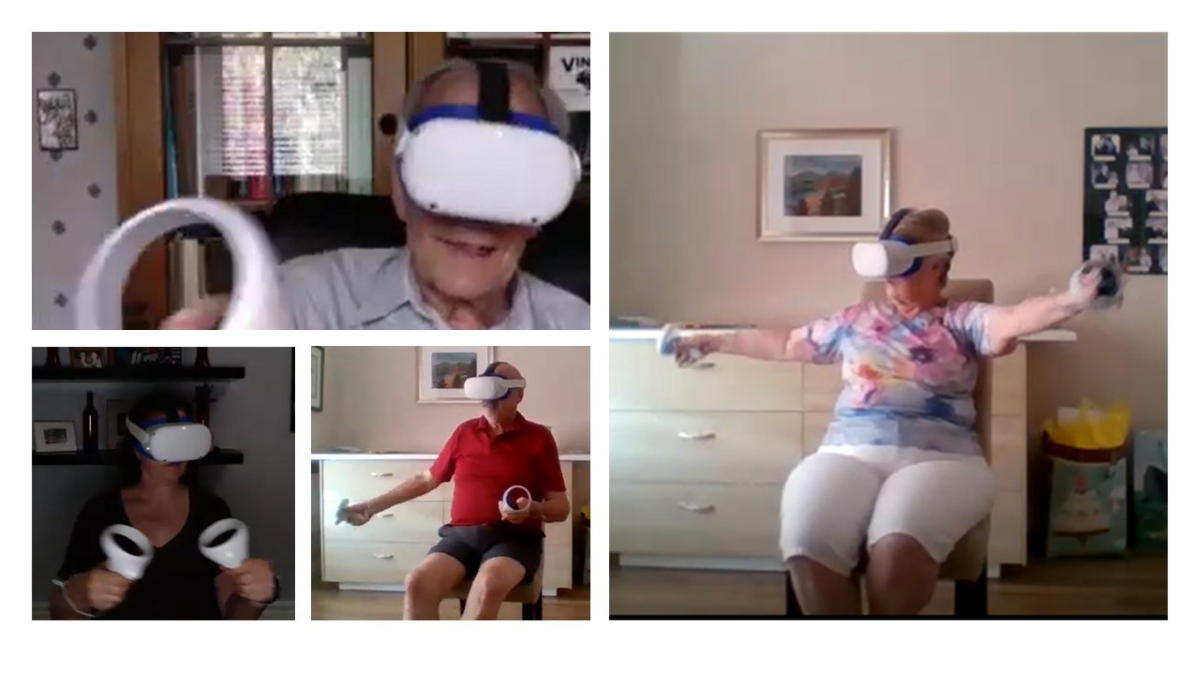
Participants playing the *Seas The Day* exergame using Oculus Quest 2 (Facebook Reality Labs).

*Seas The Day* is designed to be played in stand-alone VR headsets. In this study, an Oculus Quest 2 will be used because of its widespread availability, relatively low weight (compared with other VR headsets), and performance when running the designed game. Two main hardware modifications will be made to the headset: (1) the conventional foam cover will be replaced with a silicon cover to facilitate cleaning and sanitization of the headset and (2) the conventional strap will be replaced with a more comfortable strap (ie, Elite Strap) [62], which allows for a more simplified way to adjust the headset (Figure 2 and Multimedia Appendix 1).

### Procedure

The participants will be asked to play *Seas The Day* 3 times a week for 6 weeks. *Seas The Day* will be the only game that the participants will have access to in the provided VR headset. The participants will be encouraged to maintain consistency in engaging with the VR exergame by playing in the mornings and preferably on the same days every week. They will be notified that each exergame session will take approximately 15 to 20 minutes and encouraged to achieve a light to moderate intensity when playing the game. Setup and ongoing participation in the intervention will be supported in a number of ways. First, the participants will be provided with step-by-step software and hardware manuals (Multimedia Appendix 2). Second, each participant will meet with the study staff or trainees for a remote introductory session via a videoconference platform. The team member will show the participants how to use the system while sharing their screen so that the participants can see and become familiar with the visual information and the overall interaction with the system. The team member will also demonstrate how to calibrate the system and play the game, stage by stage, and answer any questions as they arise. The participants will then be encouraged to interact with the system and try playing the exergame in the presence of the team member and speaking aloud about what they are seeing and experiencing so they can be guided if facing any difficulties. Finally, the participants will be able to contact the study staff and trainees to troubleshoot the system via email, phone, SMS text message, or video calls at any time as most appropriate for the situation and the participant’s comfort. To facilitate troubleshooting video calls, the participants will be offered screen-sharing options to facilitate the explanation as well as a view from the frontal camera of the computer to see how the team member is located and moving in the physical space.

### Ethics Approval

This study has been reviewed and received ethics clearance through the University of Waterloo Research Ethics Committee (experimental group 42908, control group 43379).

### Data Collection and Assessments

#### Screening

The participants will complete a verbal informed consent agreement before taking part in the study and will be screened (via email or telephone) for eligibility using (1) a custom-made screening questionnaire and (2) the GAQ. The custom-made screening questionnaire is developed by the research team and includes questions on the study’s inclusion and exclusion criteria. The GAQ, developed by the Canadian Society for Exercise Physiology [56], is a prescreening measure for physical activity to ensure the participants’ safety when partaking in at-home exercise. The GAQ includes sections on preparing to become more physically active, assessing current physical activity, and general advice for becoming more active. A record of ineligible cases and those who did not provide consent to participate in the study will be kept in the screening log to inform the recruitment process. No further information will be collected from these cases.

#### Demographics

Background and demographic data will be obtained through (1) a demographic questionnaire, (2) the remote MoCA (version 8.1-Telemed) [58], and (3) the short version of the Geriatric Depression Scale-15 (GDS-15) [63]. The demographic questionnaire is developed by the research team and collects basic information of the participants (eg, age, sex, gender, and education), their health (eg, perceived physical and mental well-being, history of falls, and hearing and vision impairments), and previous experiences with gaming and motion sickness. The MoCA is a validated screening tool for cognitive impairment with high sensitivity and specificity for detecting mild cognitive impairment and dementia [57]. The MoCA will not be used for diagnostic purposes but will be used by the researchers to gain insight regarding the sample’s baseline cognition (a MoCA score of <13 suggests severe cognitive impairment; this study will use a cutoff score of ≥18 for study inclusion). The remote audiovisual administration of the MoCA will be conducted by a trained researcher. The GDS-15 is a widely used depression assessment tool that is specifically designed for older adults. Similar to the MoCA, the purpose of using the GDS-15 is to gain insight into the sample’s baseline depressive symptoms (if any). The GDS-15 consists of 15 questions about how the participant has felt over the previous week.

#### Outcomes and Measures

##### Overview

All participants will complete assessments at baseline and after the intervention (follow-up). Upon completion of the study (in a period of 8 weeks), semistructured focus groups or interviews will be conducted (with distinct questions for the control vs the experimental groups), and informal notes and reports will also be obtained from both the experimental and control groups. All participants will complete the exercise self-efficacy questionnaire; further acute changes in executive function, multisensory integration, and mood before and after gameplay or reading will also be assessed. In addition, the experimental group will complete the RPE [64] and perceived enjoyment scales upon completion of each exergaming session. All questionnaires except for RPE and perceived enjoyment for the experimental group will be completed on the web using Qualtrics survey software (Qualtrics International Inc) [65]. Refer to Table 1 for a summary of the outcomes and instruments used in this study and Table 2 for the study timeline.

**Table 1:** Summary of outcome measures and instruments.

Outcome measures		Instrument
**Primary outcome measures**		
	Background and demographic data	Demographic questionnaire MoCA^a^ GDS-15^b^ GAQ^c^
	Feasibility, usability, and accessibility of at-home VR^d^ exergaming	Self-reported physical (eg, motion sickness, vertigo, or nausea) or emotional (eg, fear or anxiety) discomfort Ad hoc usability and game user experience questionnaire Perceived enjoyment Exergaming session adherence rate Informal notes and reports Semistructured focus groups or interviews
	Feasibility of the study protocol	Consent and recruitment rate Attrition and retention rate Activity log Rate of missing data Semistructured focus groups or interviews
**Secondary outcome measures**		
	Physical activity level	PASE^e^ RPE^f^
	Executive function	Modified flanker task OTMT^g^ (part A and B) VF^h^ (animal naming)
	Multisensory integration and temporal perception	RT^i^ task SJ^j^ task SIFI^k^ TOJ^l^ task
	Affect	PAAS^m^
	Exercise self-efficacy	Bandura exercise self-efficacy scale

^a^MoCA: Montreal Cognitive Assessment.

^b^GDS-15: Geriatric Depression Scale-15.

^c^GAQ: Get Active Questionnaire.

^d^VR: virtual reality.

^e^PASE: Physical Activity Scale for the Elderly.

^f^RPE: rate of perceived exertion.

^g^OTMT: Oral Trail Making Test.

^h^VF: verbal fluency.

^i^RT: response time.

^j^SJ: simultaneity judgment.

^k^SIFI: sound-induced flash illusion.

^l^TOJ: temporal-order judgment.

^m^PAAS: Physical Activity Affect Scale.

**Table 2:** Summary of the study timeline.

Outcome measure and instrument		Time points																			
		Baseline	Week 1			Week 2			Week 3			Week 4			Week 5			Week 6			Follow-up
			Before	After	Before		After	Before		After	Before		After	Before		After	Before		After		
**Background and demographics**																					
	Demographic questionnaire	✓																			
	MoCA^a^	✓																			
	GDS-15^b^	✓																			
	GAQ^c^	✓																			
**Executive function**																					
	OTMT^d^ (A and B)	✓	✓	✓				✓		✓				✓		✓				✓	
	VF^e^	✓	✓	✓				✓		✓				✓		✓				✓	
	Modified flanker task	✓	✓	✓				✓		✓				✓		✓				✓	
**Multisensory** **i** **ntegration and temporal perception**																					
	SIFI^f^ task	✓			✓		✓				✓		✓				✓		✓	✓	
	SJ^g^ task	✓																		✓	
	TOJ^h^ task	✓																		✓	
	RT^i^ task	✓	✓	✓				✓		✓				✓		✓				✓	
**Physical activity, mood, and exercise self-efficacy**																					
	PAAS^j^	✓	✓	✓	✓		✓	✓		✓	✓		✓	✓		✓	✓		✓	✓	
	PASE^k^	✓																		✓	
	Exercise self-efficacy	✓					✓						✓						✓		
	RPE^l^			✓			✓			✓			✓			✓			✓		
	Perceived enjoyment			✓			✓			✓			✓			✓			✓		
Usability and game user experience questionnaire																				✓	

^a^MoCA: Montreal Cognitive Assessment.

^b^GDS-15: Geriatric Depression Scale-15.

^c^GAQ: Get Active Questionnaire.

^d^OTMT: Oral Trail Making Test (part A and B).

^e^VF: verbal fluency (animal naming).

^f^SIFI: sound-induced flash illusion.

^g^SJ: simultaneity judgment.

^h^TOJ: temporal-order judgment.

^i^RT: response time.

^j^PAAS: Physical Activity Affect Scale.

^k^PASE: Physical Activity Scale for the Elderly.

^l^RPE: rate of perceived exertion.

##### Feasibility of the Study Protocol and VR Exergaming

The rates of recruitment, attrition, retention, and missing data will be used to assess the feasibility of the study protocol. The feasibility, usability, and accessibility of *Seas The Day* will be assessed using quantitative and qualitative data. Quantitative data will be collected from (1) an ad hoc usability and game user experience questionnaire, (2) the participants’ adherence rate to the exergaming sessions, and (3) the participants’ perceived enjoyment. Qualitative data will be obtained from (1) self-reported physical (eg, motion sickness, vertigo, or nausea) or emotional (eg, fear or anxiety) discomfort (if any), (2) the participants’ informal notes and reports, and (3) semistructured focus groups or interviews.

The ad hoc usability and game user experience questionnaire is developed by the research team. Subscales of validated usability and acceptability models [66,67] are used in this questionnaire to explore users’ attitudes and intentions of using VR exergame systems (eg, perceived usefulness, perceived ease of use, perceived enjoyment, and intention to use in the future). The participants will be asked to rank each question from 1 to 5 based on how much they agree with each statement. The participants’ perceived enjoyment will be assessed using a paper-based, pictorial 5-point Likert scale ranging from *not enjoyable at all* to *extremely enjoyable*.

Semistructured focus groups or interviews (approximately 60 minutes) will be conducted to obtain in-depth information regarding each participant’s experience of the study procedures and measures. For the experimental group in particular, focus groups or interviews will be used to gain more insight into the participants’ perceived feasibility, acceptability, and usability of the VR exergaming system (eg, satisfaction, ease of use, engagement, safety, and motion sickness). Facilitators of and barriers to the adoption of and adherence to the VR exergaming system will also be explored in focus groups or interviews. For the control group, the focus groups or interviews will be used to gain insight regarding technology use and adoption and obtain feedback on the web-based tasks and assessments.

##### Executive Function

Measures of executive function will consist of a web-based modified flanker task, the Oral Trail Making Test (OTMT) parts A and B, and a verbal fluency (VF) test (animal naming). The modified flanker task includes a set of response inhibition tests to assess the ability of the participants to suppress responses that are inappropriate in a particular context [68,69]. In total, 3 types of stimuli—incongruent, congruent, and neutral—will be represented by arrowheads. In all sessions, the participants will be asked to complete 4 blocks: 1 practice block consisting of 30 mixed stimuli (10 experiments per flanker arrow type) to help familiarize the participants with the task, 2 blocks consisting of 100 trials (20 incongruent and 80 congruent flanker arrows [70]), and 1 block consisting of 100 trials (all neutral flanker arrows). The display duration of the flanker arrows will be 150 ms, and the intertrial interval duration (from a + sign to the next) will be 1500 ms (−100 to +100 ms). The participants will be asked to sit in a quiet room while directly facing their computing device. They will be instructed to keep their index fingers on the right and left arrow keys of their computer keyboard (to indicate the direction of the flanking arrow), look at a small white fixation cross in the middle of a black screen where the target stimuli will appear, and respond as quickly and accurately as possible. The task will take approximately 10 to 15 minutes to complete, and the participants will be able to take a break in between blocks if they wish. The OTMT is a neuropsychological test and can reflect a wide variety of cognitive processes [71-73]. The test consists of 2 conditions: condition A, in which the participants are instructed to count from 1 to 25, and condition B, in which the participants are instructed to count in an alternating numeric and alphabetic sequence (ie, 1-A-2-B). The goal of the test is for the participant to finish both parts as quickly as possible, and the time taken to complete the test will be used as the primary performance metric. The OTMT takes approximately 2 to 5 minutes to complete. VF is a widely used test of executive function in which the participants will be given 60 seconds to produce as many unique words as possible within a semantic category (ie, names of animals) [74,75]. The number of unique words will be the participant’s score on this task. Both the OTMT and VF will be administered in an oral format via phone or an audio- and videoconferencing platform with the guidance of a trained researcher.

##### Multisensory Integration

Four web-based perceptual tasks will be performed: (1) the audiovisual response time (RT) task, (2) the sound-induced flash illusion (SIFI), (3) the simultaneity judgment (SJ) task, and (4) the temporal-order judgment (TOJ) task. The audiovisual RT, SJ, SIFI, and TOJ tasks have been specifically designed for web-based data collection. These tasks will be used to obtain various measures of sensory integration, including RT (to assess RT differences between uni- and multisensory cues), temporal binding window (TBW; the window of time during which stimuli from different modalities are bound together), and susceptibility to the SIFI (where the observer misperceives the number of visual flashes owing to the presentation of 2 auditory beeps in close temporal proximity).

The participants will be asked to sit in a quiet, dark room while directly facing their computing device, which will be placed at arm’s length. The visual stimuli will be presented in the form of white circles, subtending approximately 2° of visual angle. The visual stimuli will appear approximately 8° below the fixation cross (visual angle=1.5°), which will appear at the center of the screen and will remain on display throughout the trial for 16 ms. The participants will be asked to fixate on the fixation cross throughout the duration of each task. Auditory stimuli will be presented in the form of beeps (16 ms) through speakers that are either connected to the participant’s device or through external speakers placed beside the screen. To reduce temporal predictability, each trial will begin with the stimulus being presented after a delay of 1000 to 3000 ms. A computer keyboard will be used by the participants to input their responses for each trial; a response from the participant will initiate the next trial for each task. The participants will complete the RT, SJ, SIFI, and TOJ tasks in a randomized order where they will be explicitly asked to respond as accurately as possible as opposed to responding quickly for the SJ, SIFI, and TOJ tasks while responding as quickly as possible for the RT task. The participants will be presented with practice trials before the commencement of each of the experimental tasks. The following stimulus onset asynchronies (SOAs; the amount of time between the start of one stimulus, S1, and the start of another stimulus, S2) will be used for the multimodal conditions in the SJ, TOJ, and SIFI tasks: 0 ms, −70 to +70 ms, −150 to +150 ms, and −230 to +230 ms; here, *+* indicates vision-led trials, whereas − indicates auditory-led trials.

For the SIFI task, there will be 3 conditions: vision-only, auditory-only, and audiovisual. In the vision-only block, 2 flashes will be presented, and the participant’s task will be to indicate the number of flashes they saw. In the auditory-only block, 2 beeps will be presented, and the participants will be asked to indicate the number of beeps they heard. There will be 30 trials in each of the unimodal conditions (SOAs: 70 ms, 150 ms, and 230 ms). The audiovisual trials will consist of 2 control conditions (1 beep and 1 flash and 2 beeps and 2 flashes) as well as the illusion condition (2 beeps and 1 flash). In the audiovisual control conditions, the auditory and visual stimuli will be presented simultaneously. In the 2 beeps and 1 flash (illusory condition) auditory-led trials, the auditory stimulus will be presented first, after which the auditory and visual stimuli will be presented simultaneously following a variable SOA. In the 2 beeps and 1 flash vision-led trials, the first auditory stimulus will be accompanied by a visual stimulus, and the second auditory beep will follow a variable SOA. The 3 audiovisual conditions will be randomly presented within the testing block to avoid response bias. The participants will be asked to fixate on the fixation cross for the duration of the task, report the number of flashes seen, and ignore the auditory stimuli. All conditions will be repeated 10 times for a total of 100 trials (including 10 repetitions for 0 SOAs, where a single beep and flash will be presented simultaneously). In total, 160 trials, as well as additional practice trials, will be presented for all 3 conditions (vision-only, auditory-only, and audiovisual). This task will take approximately 5 to 10 minutes to complete.

For the SJ task, the participants will be instructed to report, using the number *1* and *2* keys on their keyboard, whether they perceived the auditory and visual stimuli as occurring simultaneously (number *1* key) or not (number *2* key). A total of 10 trials will be presented in a randomized order for each SOA, and 6 practice trials will also be presented for a total of 76 trials. This task will take approximately 5 to 10 minutes to complete. The experimental design of the TOJ task will be identical to that of the SJ task with the exception of the task instructions. In this case, the participants will be asked to report, using the number *1* and *2* keys on their keyboard, whether they perceived the visual (number *1* key) or auditory (number *2* key) stimulus as appearing first.

For the RT task, the participants will be told that they will see a flash of light, hear a beep, or a combination of the 2, and they will be instructed to press the response button (space bar key) as soon as they detect any of the 3 experimental conditions. Each condition will be presented 50 times in a random order with 6 practice trials for a total of 156 trials. However, if a participant responds too quickly (<100 ms) or takes longer than 3 seconds to respond to a trial, that trial will be repeated. This task will take approximately 5 to 10 minutes to complete.

Both the multisensory integration and modified flanker tasks will be designed using PsychoPy (Open Science Tools Ltd) and distributed on the web using Pavlovia (Open Science Tools Ltd), wherein the participants will be provided with URLs to access the tasks. All instructions will be embedded within the tasks and will not require the presence of a researcher to be completed. However, a researcher will join the participants for the duration of the first session of these tasks to ensure that all inquiries and technical difficulties are resolved as soon as they arise. Furthermore, 1 to 2 members of the research team will check in with the participants every 2 weeks to answer any questions and ensure that they are following the protocol.

##### Physical Activity, Exercise Self-efficacy, and Mood

The Physical Activity Scale for the Elderly (PASE) [76] and RPE will be used to assess the participants’ physical activity behavior. In the PASE, the participants will be asked to self-report the frequency with which they take part in leisure, household, and occupational activities (eg, outdoor walking; light, moderate, and strenuous sports and recreation; and muscle strengthening) by indicating never, 1 to 2 days per week (seldom), 3 to 4 days per week (sometimes), or 5 to 7 days per week (often). Activity duration will be indicated as <1 hour, between 1 and 2 hours, 2 to 4 hours, or >4 hours. RPE will be assessed through a paper-based pictorial omnibus scale wherein the participants will be asked to indicate how physically exhausting the VR exergaming was using a 10-point Likert scale ranging from *extremely easy* to *extremely hard* [64].

Mood will be assessed using the Physical Activity Affect Scale (PAAS) [77]. Affective states are thought to be highly influenced by exercise, thus leading to the development of the PAAS as a concise affect measurement tool [77-79]. The PAAS has 12 items, which can be further broken down into 4 subdomains, each further broken down into 3 mood states: positive affect (upbeat, energetic, and enthusiastic), negative affect (miserable, discouraged, and crummy), physical exhaustion (tired, worn-out, and fatigued), and tranquility (calm, peaceful, and relaxed). Exercise self-efficacy will be assessed using the Bandura exercise self-efficacy scale, which is a predictor of the adoption and maintenance of exercise behavior [80]. The Bandura scale includes 9 statements, and the participants will be asked to rank each statement from 1 to 4 indicating their belief in their ability to continue exercising in the future.

##### Automatic Data Logging System

*Seas The Day* includes a data logging system that allows for the capture of data related to game events and interactions inside the virtual environment during gameplay. In every session, the data logging system locally records data using a time series at 30 Hz combining game events, scores, achievements, and kinematic information from the VR equipment (controllers and headset). The data logging system records the following information: (1) tai chi (time to complete the activity and interactions with animals [when players were looking at them]), (2) rowing (boat speed, distance traveled, number of strokes [right or left], boat collisions, and interactions with the dolphin), and (3) fishing (number of fish caught, pulling out the fishing rod [number of repetitions], and RT after the fish is hooked). The kinematic information is captured through the inertial motor units embedded in the 2 VR controllers and the headset, recording 3-axis accelerometer information of the 3 points (head and right and left hand). Both game variables and kinematic information are locally stored in the headset and compressed in files of 15 MB.

### Statistical Analysis Plan

#### Sample Size

Given that the primary purpose of the study is to assess the feasibility and usability of at-home VR exergaming over a 6-week period, no formal sample size calculation will be performed [81]. However, to explore data collection processes and also inform sample size calculations for a larger trial, the study will aim to recruit at least 12 participants for both the experimental and control groups [82].

#### Feasibility Analysis

Feasibility will be assessed through the recruitment rate (number of participants divided by the total number of potentially eligible participants), attrition rate (percentage of participants who did not complete the study), and adherence rate (number of completed exergaming or reading sessions divided by the total number of potential exergaming or reading sessions). Appropriate qualitative analyses will be carried out to provide further details regarding the feasibility, usability, and acceptability of the VR exergame as well as the study protocol. The focus group or semistructured interview script includes open-ended questions (Multimedia Appendix 3). These questions were refined through pretesting with a volunteer group of older adults (n=5) to ensure clarity and prompt discussion relevant to the issues of immediate interest [61]. Using a focus group allows the participants to exchange viewpoints and engage in conversations with peers to express general agreement or disagreement on various aspects of our study [83,84]. Individual interviews, by contrast, allow for a deeper understanding of the participants’ experiences, beliefs, and attitudes through direct, one-on-one engagement [85]. Both methods will be considered in our study; however, deciding on whether to participate in a focus group or a one-on-one interview (or both) will be based on the participants’ preferences and what would be the most feasible for them.

Focus groups or interviews will be digitally recorded (via videoconferencing platforms) and transcribed verbatim. All qualitative data (ie, focus groups or interviews, self-reported physical or emotional discomfort, and the participants’ informal notes and reports) will be organized and coded using NVivo (version 12; QSR International) [86]. Inductive and deductive thematic analyses (informed by the feasibility, usability, and acceptability objectives of the study) will be performed after line-by-line coding to identify key topics and patterns of meaning across the data [87-89]. Open coding and axial coding will be conducted by multiple researchers independently to enhance the rigor of data analysis [89-91]. Researchers will use reflexive memos during the coding process to reflect on the emerging themes and patterns [87,92,93]. To create a robust codebook, researchers will regularly meet to resolve any coding challenges or discrepancies. The codebook will be refined by discussing the coding scheme (as different key topics emerge) and merging or deleting overlapping codes. After a consensus is reached, the codebook will include definitions and examples of each code, and the coding process will continue until data saturation (ie, no new data and no new themes are observed) [94,95]. Codes will then be categorized to detect overarching themes as well as subcategories to support themes [87].

#### Outcome Analyses

Descriptive statistics will be calculated for all demographic and baseline characteristics of the participants and presented as means and SDs or percentages (n), as appropriate. Assumptions underlying the statistical procedures will be checked, and corrective procedures will be used if necessary. Statistical analyses will be performed in R Studio (R Foundation for Statistical Computing) and SPSS (IBM Corporation). Data from the cognitive and perceptual tasks will be assessed for normality using *Q*-*Q* plots, the Shapiro-Wilk test, and histograms. Homogeneity of variance will also be tested using the Mauchly sphericity test, and Greenhouse-Geisser corrections will be applied if necessary. Potential deviations from a normal distribution will be identified in all cognitive and perceptual task data sets, and log transformations will be applied to the raw skewed data if deemed necessary to transform the data into a normal distribution. Paired and independent 2-tailed *t* tests will be used to compare the baseline, follow-up, and acute scores. Repeated-measure and mixed ANOVAs will be used to analyze before and after intervention data (exergaming or reading for 6 weeks) as well as acute data (before and after 1 bout of exergaming or reading) to understand changes by time (before and after), group (control vs experimental), and day (eg, weeks 2, 4, and 6 for SIFI) to compare baseline, follow-up, and acute scores.

#### Executive Function

Cognitive data from the OTMT (completion time [seconds] for parts A and B separately) and VF (the number of unique animal names) will be analyzed for within-participant and between-participant variables. For the modified flanker task, the participants’ mean RT and proportion error (1-accuracy) for each block of flanker responses will be obtained and further separated by congruence (congruent, incongruent, and neutral). Incorrect responses, responses that occurred 1000 ms after stimulus onset, and double responses for a single stimulus will be considered as errors and excluded from the analysis. The rate of no response and gender will be included as covariates.

#### Multisensory Integration

##### SIFI Task

Analyses will be conducted to determine whether there are differences in before and after intervention (exergaming or reading for 6 weeks) data as well as for before and after acute (1 bout of exergaming or reading) data. Analyses will be conducted separately on the proportion correct for unimodal (modality: vision-only or auditory-only) and multimodal (audiovisual conditions: nonillusory, 1 flash and 1 beep or 2 flashes and 2 beeps; or illusory, 1 flash and 2 beeps) conditions. To examine changes in susceptibility to the SIFI, sensitivity (*d*) will be calculated (*z*[hits] − *z*[false alarms]) for each SOA, and appropriate analyses will be conducted [96-98].

##### SJ and TOJ Tasks

To estimate the accuracy (point of subjective simultaneity) and precision (TBW) with which the participants make their judgments for the SJ and TOJ tasks, psychometric functions will be fitted to each participant’s responses as a function of SOA. To investigate the relationships between TBWs obtained from the 2 tasks and not their absolute size, the *b* values (ie, SD) of these psychometric functions will be analyzed as a proxy for the size of the TBW to avoid discrepancies in the literature that differ when defining the absolute size of the TBW [99-101]. Each task will be analyzed individually for each participant, with participant data fitted to both Gaussian and logistic functions.

##### RT Task

Data trimming procedures will not be applied [99,102]; however, responses faster than 100 ms and slower than 1500 ms will be set to infinity rather than excluded [99,103] where this method of data trimming was used. The mean RT for each modality (auditory, visual, or audiovisual) before and after the experimental and control conditions will be calculated individually for each participant, and appropriate analyses will be conducted to determine the impact of time and modality. Acute data (before and after exergaming or reading) will also be analyzed as described previously.

#### Physical Activity, Exercise Self-efficacy, and Mood

The responses to the structured surveys will be cleaned and analyzed using appropriate statistical methods. Data from web-based questionnaires and surveys will be classified and analyzed using Qualtrics software [65]. In addition, PASE scores will be calculated before and after experimental (exergaming) or before and after control (reading) conditions and analyzed using paired or independent 2-tailed *t* tests. Responses to the PAAS will be classified into one of the 4 affective subscales: positive affect, negative affect, physical exhaustion, and tranquility [77]. Scores within each subscale will be treated as independent outcomes to identify acute changes in affective states from before to after experimental and before to after control conditions. RPE, perceived enjoyment, exercise self-efficacy, and PAAS will be analyzed to determine potential changes over time (before vs after experimental or control conditions) and across conditions (eg, subscales and intensity).

#### Gameplay Metrics

Game metrics captured by the data logging system will be analyzed session by session, and specific metrics associated with game performance, errors, and RT will be extracted from the described variables. First, changes in SD units for each session will be determined for 3 main variables denoting game performance on each game activity: time to complete the tai chi condition, distance traveled during the rowing condition, and the number of fish caught in the fishing condition. The number of collisions will be identified as a potential variable to quantify errors during rowing. RT will be computed in the fishing condition, where the time taken by players to pull out the fishing rod after feeling the fish being hooked (controllers vibrating) will be extracted from the data files. Time will then be filtered, and values outside the 100 to 1000 ms range will be discarded [104]. All numerical data will be presented as means and SDs.

#### Exploratory Analysis

To better understand the relationship between lower-level perceptual processing and multisensory integration (assessed via the RT, SIFI, SJ, and TOJ tasks) and higher-order executive function (assessed via the modified flanker task, VF, and OTMT), exploratory correlational analyses will be conducted between the outcomes of each of the perceptual tasks (eg, TBW, point of subjective simultaneity, mean RT from the multisensory and unisensory conditions, and accuracy) and the outcomes of the modified flanker task (accuracy and mean RT), VF, and OTMT. Furthermore, exploratory analyses using correlations are planned using both game metrics and cognitive assessments (ie, modified flanker and RT tasks) for RT. Partial correlation analyses, including age, sex, and previous experience with video games as covariates, will be applied when appropriate. Correlational analyses will be conducted for outcome variables obtained before and after 6 weeks of the exergame intervention and before and after acute bouts of engagement with the exergame. The Bonferroni correction will be used to correct for multiple comparisons.

#### Handling of Missing Data

Any missing data will be reviewed and imputed based on the assessments that the participants complete on other days. If the missing data of a variable do not depend on the value of another variable, the analysis will be performed using both the complete data set and an analysis incorporating values from multiple imputations. If the missing data are not random, then the analysis will be completed using only the complete data set; however, differences between the 2 groups with missing and observed data will be reported.

## Results

This study aims for an estimated adherence rate of ≥80%. To minimize potential attrition, all participants will be provided with a thorough remote one-on-one explanation of the anticipated participation burden (time commitment) at baseline. The participants will also be contacted regularly throughout the data collection process to maintain a good working relationship and answer any questions that they may have. The participants may choose to withdraw from the study at any time and for any reason. However, they will be asked if their collected data can be used by the research team for information and data analysis purposes.

We anticipate that most of the enrolled participants will adhere to the study and follow the protocol. However, potential challenges related to nausea and motion sickness when interacting with the VR technology may lead to some dropouts in the experimental group [105-108]. Challenges related to remote assessments as well as the length and complexity of the cognitive and multisensory integration tasks may also result in attrition [109-112].

In addition, we anticipate that most participants will learn how to navigate virtual environments and successfully perform the proposed physical activities within the different stages of our exergame [113,114]. This can potentially lead to high levels of perceived enjoyment and study adherence, as found in similar studies using immersive VR with the same duration and frequency [115]. However, it is also expected that some older adults might need more support than others to set up the VR system and adapt to the technology [113,114,116,117]. These supports might need to be individualized and tailored as the pilot progresses.

Both the experimental and control conditions have been reviewed and received ethics clearance through the University of Waterloo Research Ethics Committee (experimental condition ethics clearance in April 2021 and control condition ethics clearance in June 2021). The screening and recruitment process for the experimental group started in May 2021, and the data collection process will be completed by September 2021. As of August 16, 2021, 15 community-dwelling older adults have enrolled in the experimental group, of whom 2 (13%) have dropped out. The recruitment process and data collection for the control group will start in September 2021 and be completed by December 2021.

## Discussion

### Overview

The use of immersive VR for promoting exercise among older adults is an emerging field of research with special relevance because of the COVID-19 pandemic. Social distancing measures and the closure of exercise facilities have been creating a more demanding need for effective home-based programs to encourage physical activity participation and enhance older adults’ well-being. This pilot study is a result of the need to move to remote research following pandemic restrictions and rapid technological advancements and adoption.

This study aims to assess the feasibility of exploring changes in perceived levels of physical activity, exercise self-efficacy, mood, executive function, and multisensory integration. Although some studies have been validated for at-home remote administration [47,52], this approach is still not commonly used and has not yet been used in conjunction with VR exergames. Challenges related to remote research, unstable internet connection, monotony with regard to multiple sessions, computer use, human errors, and eye strain are expected during remote testing [110,111,118-120]; part of this pilot is to hone ways to mitigate and solve these challenges remotely. This pilot will add insights into the feasibility of implementing a relatively complex, remotely administered assessment protocol for older adults living at home.

This protocol also addresses unexplored research questions regarding the use of state-of-the-art VR technologies and custom-made exergames tailored to older adults for home-based programs. First, this study will examine the feasibility of deploying exercise programs using immersive VR with *Seas The Day* during the COVID-19 pandemic. The study intervention has a longer duration than the average found in similar studies of the same nature [47,52]. Other studies using immersive VR in older adults have exposed participants to VR experiences ranging from 1 to 55 minutes, with 15 minutes being the most common exposure time [47,121,122]. Although many elements are included in this study to support community-dwelling older adults to easily set up and use the VR system at home, challenges associated with system calibration, the overall use of the tracking system, and potential motion sickness, among others, are to be expected during the study [105-108,113,114].

The deployment of VR systems at home also has the novel potential to capture data during gameplay, which may be able to capture changes not only in game performance but also in older adults’ well-being. In this study, the VR system will capture a broad range of variables that will be used to recreate the microinteractions and behaviors of the participants during their interaction with *Seas The Day.* Although exploratory, previous work has shown how gameplay metrics have the potential to be powerful descriptors of elements related to physical and cognitive functioning, which are very important aspects when tracking the progress and benefits of exercise programs [121,122].

### Strengths and Limitations

This study will establish the feasibility of deploying a VR system to older adults across Canada for health application purposes. This study will also assess the feasibility of a complex assessment protocol. Both may provide useful insights for the future of remote, virtual research and are specific not only to VR research but also more broadly applicable.

Reaching our target population may be challenging during the COVID-19 pandemic and will likely be restricted to older adults who are open to the use of technology and have the ability to do so (eg, have a computer or tablet and internet already). In addition, older adults may be hesitant to participate because of (1) technology apprehension, (2) availability of other physical activity options (as, at the time this paper was written, the restrictions had started to loosen up), and (3) time commitment (8 weeks total, with multiple tests and web-based meetings). We plan to minimize the impact of these limitations by carrying out introductory sessions to facilitate the usability of the technology at the beginning of the trial, using stand-alone VR systems that are portable and can be used without an internet connection, and building rapport with all the participants to facilitate a long-standing research relationship and reduce the burden of taking part in this research remotely.

Another important limitation associated with this pilot study protocol is a lack of control over the VR exergame and assessment environment. Although we will strongly suggest that the participants engage with the exergames in the mornings and preferably on the same days every week, we cannot ensure that these guidelines will be followed. Unlike tasks administered in a well-controlled laboratory environment, the cognitive and perceptual tasks will be administered to the participants in the comfort of their homes through a web-based platform, which reduces our control over the size, brightness, and intensity of the stimuli. Furthermore, variations in the size and positioning of the monitor and keyboard as well as the placement of the speakers further decrease experimental control. The participants will be encouraged to maintain their environment (eg, use the same monitor and stay in the same room) once they complete their first session of cognitive and perceptual tasks, establishing consistency within the participants. Although these challenges may make collecting data more difficult, they also represent data that are much closer to real-world everyday use than they would be in a laboratory environment.

Finally, the small sample size of this pilot study and the disadvantages associated with self-reported questionnaires (eg, inaccurate data and social desirability bias) limit the generalizability of the results and, thus, the statistical analyses must be interpreted with caution [123,124].

### Dissemination

Dissemination of the results will be led by the authors in a collaborative effort and will include (1) presentations at international conferences and publications in scientific peer-reviewed journals on topics related to active aging, older adults’ physical and mental well-being, VR exergaming, or other similar areas of interest; (2) reports to the study stakeholders (eg, the VR company); (3) webinars or similar for the general public (including older adults); and (4) information sessions with exercise professionals in long-term care facilities and retirement homes to explore potential applications of *Seas The Day* as a viable way to foster physical activity in their settings.

### Conclusions

This pilot study will provide insights into the feasibility, acceptability, and usability of a custom-made VR exergaming intervention to promote at-home exercise in older adults, which may potentially benefit their physical and mental well-being. Integrating qualitative and quantitative methods will help identify potential barriers to and facilitators of home-based VR exergaming and explore older adults’ perspectives and required accommodations to support their uptake. Findings from this feasibility study will also be useful to inform the methodology, design, study procedures, and assessment protocol for future large-scale trials of VR at home with older adults. It is anticipated that the results from this pilot can be used by others to support the design and deployment of other remote exergames and assessments.

## Appendix

Multimedia Appendix 1 COVID-19 virtual reality exergames standard operating procedures.

Multimedia Appendix 2 Virtual reality exergame manual.

Multimedia Appendix 3 Interview and focus group guide (experimental and control groups).

## Abbreviations

GAQ: Get Active Questionnaire
GDS-15: Geriatric Depression Scale-15
MoCA: Montreal Cognitive Assessment
OTMT: Oral Trail Making Test
PAAS: Physical Activity Affect Scale
PASE: Physical Activity Scale for the Elderly
RPE: rate of perceived exertion
RT: response time
SIFI: sound-induced flash illusion
SJ: simultaneity judgment
SOA: stimulus onset asynchrony
TBW: temporal binding window
TOJ: temporal-order judgment
VF: verbal fluency
VR: virtual reality
