# Supplementary material for: Immersive Virtual Reality Exergames to Promote the Well-being of Community-Dwelling Older Adults: Protocol for a Mixed Methods Pilot Study
Source: JMIR Res Protoc. 2022 Jun 13;11(6):e32955. doi: 10.2196/32955 (PMC9237784; doi:10.2196/32955)
Supplement: Multimedia Appendix 1 [file resprot_v11i6e32955_app1.pdf]

Appendix A.1

COVID19 - VR Exergames Standard Operating Procedures

Purpose and Background

The purpose of this protocol is to outline the safety and hygiene procedures that will be taken when conducting the research study . This protocol has been prepared according to the following documents: i) healthy safety guidance during COVID19, University of Waterloo (link), ii) proceedings of the FDA public workshop "Medical extended reality: toward best evaluation practices for virtual and augmented reality in medicine" (link), and iii) recommendations from the Academy of International Extended Reality "COVID-19 Safety for virtual & augmented reality AIXR Guidelines" (link). Moreover, this protocol has been created considering procedures and activities that are similar to the ones implemented to disinfect and clean non-critical medical equipment (e.g., electrocardiography machines) in long-term care facilities (link).

The following documentation will be used by one of the members of the research team in charge of sanitizing the VR equipment and delivering it to the participants.

VR Equipment that will be used in the study

- **VR system – Oculus Quest 2**
    - Parts:
        - Head-mounted display and strap

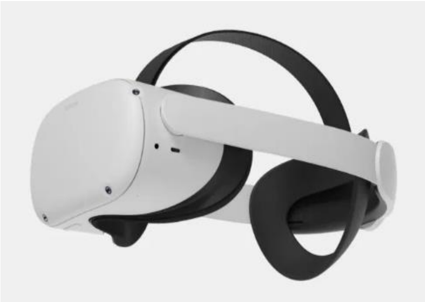

        - Controllers

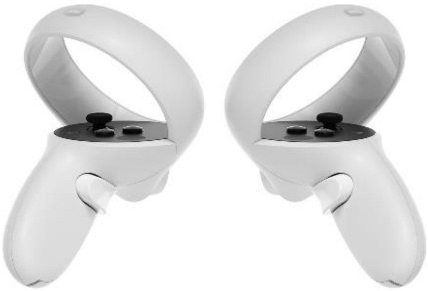

Necessary Hardware for Hygiene and Safety Considerations

- **Replacement of cover inside of the head-mounted display**
  The porous foam material placed on the inside part of the head-mounted display must be replaced as the current one allows bodily fluids to seep into the material, and thus may reduce the effectiveness of the cleaning procedures. To avoid this issue, a silicon cover for the Oculus Quest headset will be used to replace the foam cover; the silicon cover is anti-sweat and allows easy cleaning (Figure 1). For the instruction on how to replace the foam with the silicon cover, please watch this video (for future references).

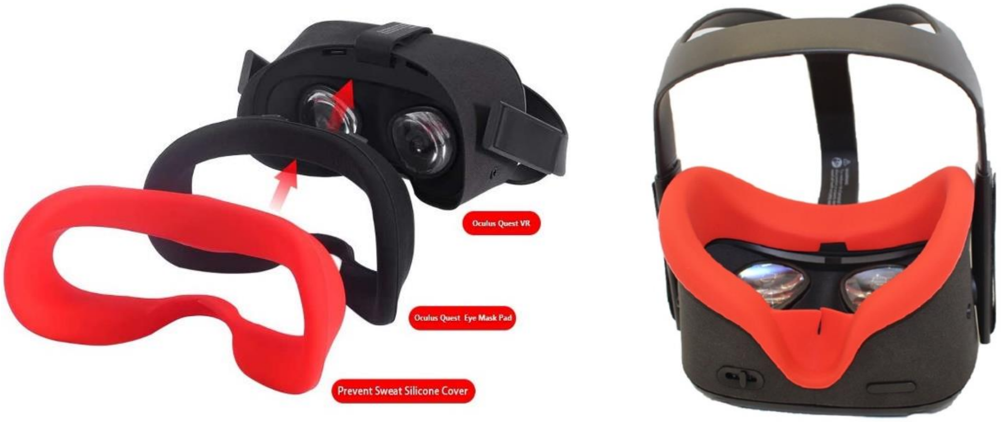

Figure 1. Silicon cover used to replace the foam cover in the Oculus Quest VR headset.

Cleaning and disinfection protocol for use of VR equipment

- **Gloves**
  - Personnel should use disposable surgical gloves or medical examination gloves when manipulating the VR headset.

- **Hand hygiene**
  Hand hygiene is the most important factor in preventing the transmission of germs/microorganisms and should be performed before and after touching VR equipment.
  - Hand washing: i) hands must be washed and/or sanitized before beginning to use VR headset, ii) hands must be washed and/or sanitized after removing gloves and after performing any surface decontamination. Resources of Public Health Ontario: how to hand wash video.
  - Hand sanitizing: all personnel trained to handle VR equipment should have alcohol-based (60% - 80%) hand sanitizer. Beyond this, frequent wipes on hands and wrists are recommended to ensure thorough sanitizing.

- **Cleaning/disinfection of headset and controllers**
  Like many other handheld devices, VR controllers can harbor germs. VR controller´s surface must be either i) wiped down thoroughly using a soft, non-abrasive damp towel/cloth soaked

with 60% - 80% alcohol solution (e.g., isopropyl alcohol), OR ii) sprayed using a 60% - 80% alcohol solution (e.g., isopropyl alcohol) onto the surface, allowing it to sit for 5 minutes, then wipe the surfaces down using a damp disposable soft, non-abrasive towel/cloth before and after using them. Make sure no fire source, including cigarettes, is around when spraying alcohol because it is highly flammable. To avoid damage and/or streaking the lenses (which will cause problems with viewing images), special care should be used when cleaning the lenses on the headset by avoiding spraying alcohol directly on their surface and wiping them down without pressure.

- **Save the headset and controllers inside the cover**
  After cleaning the VR equipment, both headset and controllers should be securely stored in the case provided to avoid unnecessary exposure.

VR equipment MUST be thoroughly cleaned and disinfected by i) spraying an alcohol solution onto the surface, leave it for 5 minutes to allow vapors to dissipate, and then ii) wiping down with a wet disposable towel before giving it to another team member. It is recommended to leave the equipment in the box for at least three days (72 hours) prior to passing it to another participant.
