# Supplementary material for: Immersive Virtual Reality Exergames to Promote the Well-being of Community-Dwelling Older Adults: Protocol for a Mixed Methods Pilot Study
Source: JMIR Res Protoc. 2022 Jun 13;11(6):e32955. doi: 10.2196/32955 (PMC9237784; doi:10.2196/32955)

VR-AT-HOME EXERGAME STUDY

Welcome to VR-At-Home project! We thank you and we are excited for you to be a part of our research.

Sincerely, the Research Team at University of Waterloo

2021

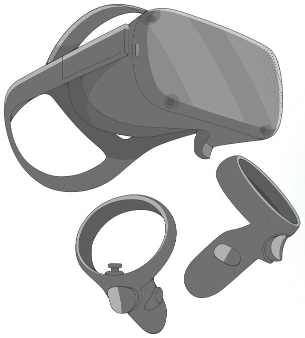

TABLE OF CONTENTS

EQUIPMENT CHECKLIST.. 1
VR at home package
General Notes

HARDWARE MANUAL.. 2
Charging the headset
Turning on/off and Restart
Using the controllers
Changing controller batteries
Cleaning the Headset/lens
Putting away Headset

SOFTWARE MANUAL.. 5
Getting ready to play
Setting up Guardian Boundary
Starting up Seas The Day
Seas The Day Gameplay
Exiting Seas The Day

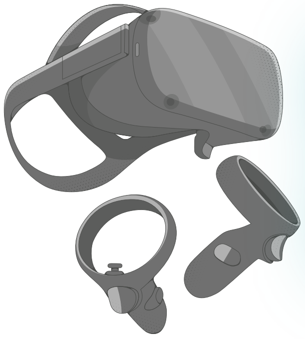

EQUIPMENT CHECKLIST

VR AT HOME PACKAGE

	Received	Packed
Oculus Quest 2 Case		
Headset	☐	☐
R/L Controllers	☐	☐
USB C Charging cable + plug	☐	☐
Batteries	☐	☐
Envelope	☐	☐
VR Manual	☐	☐
Intervention Booklet	☐	☐

GENERAL NOTES

- We ask that **only participants involved in the study** can use the VR Headset, and to only use it for the research study.
  - This is for safety reasons and to avoid affecting the data collection.
- Play the game only **seated** on chair with no arm rests and spacious environment.
- If you are experiencing any adverse reactions of motion sickness, extreme headaches and/or dizziness at any point while using VR please remove the headset and rest until you feel well.
- Take breaks during sessions if needed to reduce likeliness of discomfort.
  - Please bring this event up with the researchers via email or phone.

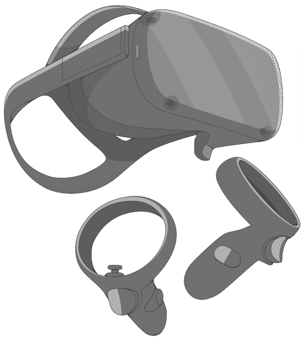

HARDWARE MANUAL

CHARGING THE HEADSET

We ask that you charge the headset after each session/use with the provided USB C charging cable.

- Plug into the left side of the headset.
- Check to see if a light appears on the right side of the headset next to the power button.
  - Orange light = charging
  - Green light = finished charging

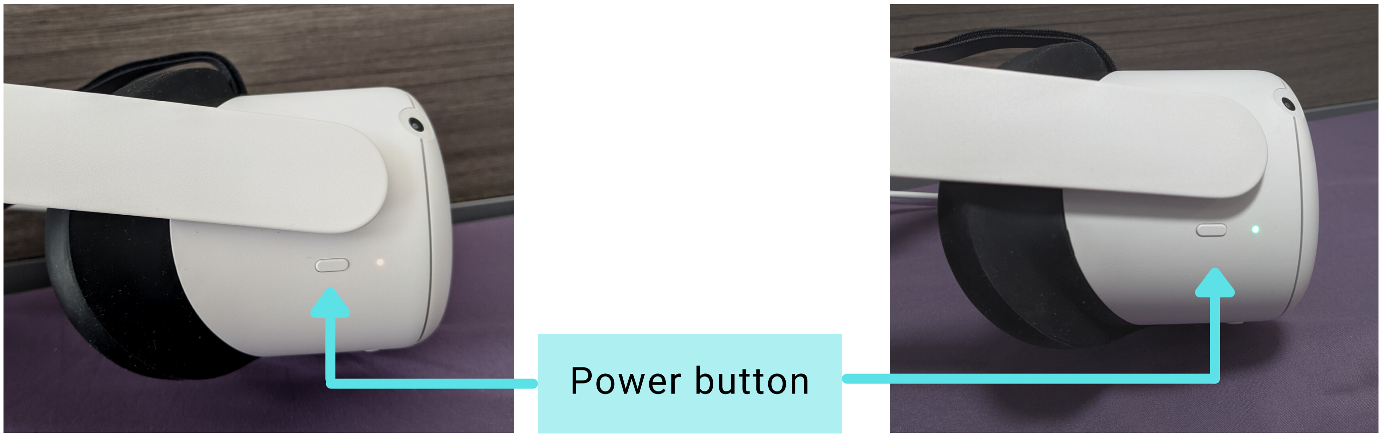

TURNING ON/OFF AND RESTARTING

- Headset should automatically turn on when placed on your head.
  - If not, press the power button located on the right side of headset
- Headset will turn off automatically after 15 seconds of inactivity
- To restart the headset, press and hold the power button for 4-5 seconds.
  - A menu will appear to restart or shut down the headset. Use the controller to select *"Restart"*

USING THE CONTROLLERS

R/L stickers are indicated on the sides of the controller to distinguish which controller is for which hand.

When using your controller, you will see a laser like beam shooting out. This will act as a cursor to select things.

- To select things, point your controller towards what you want to select and press the button where your index finger is (may also be known as your trigger finger).

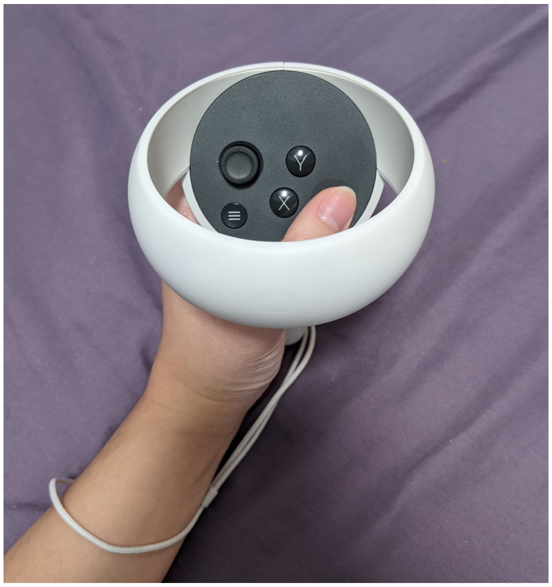

Left hand

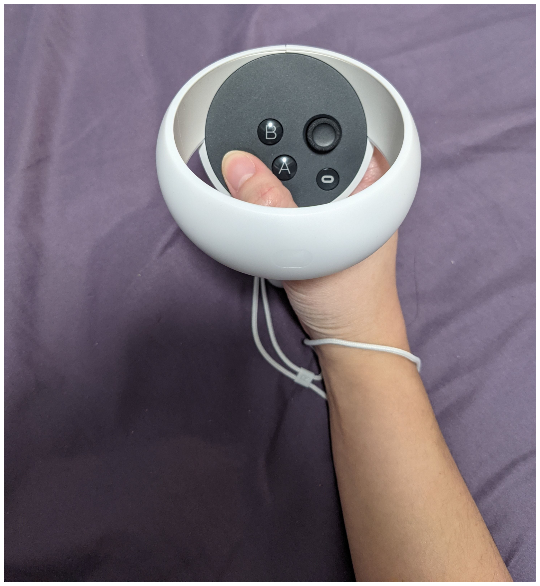

Right hand

Select with this button (trigger button)

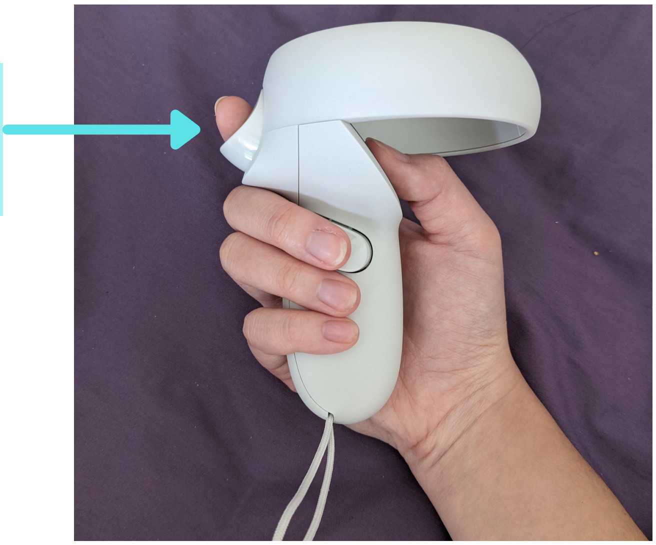

Sideview of how to hold the controller

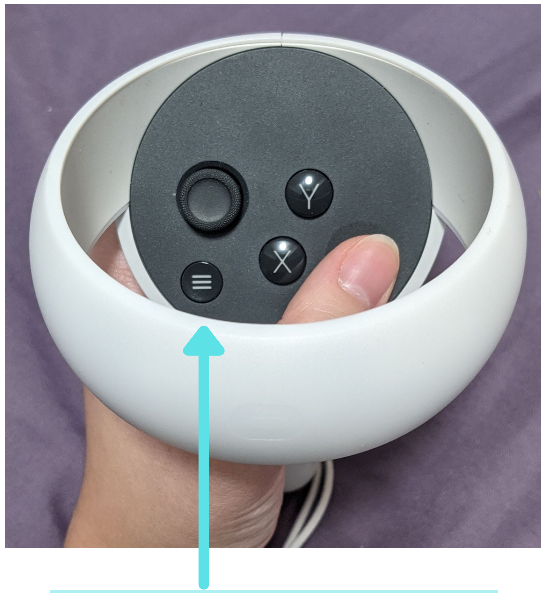

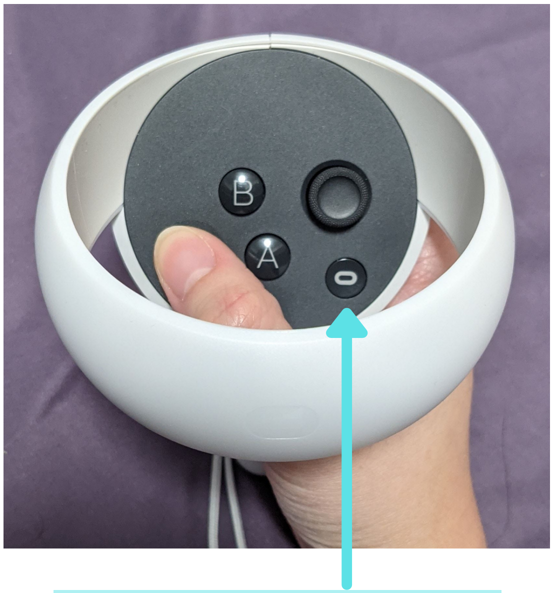

Options button (looks like 3 horizontal lines)

Oculus button (looks like a horizontal 0)

CHANGING CONTROLLER BATTERIES

There will be batteries in the controller and extra AA batteries provided for you in case the system asks you to replace the batteries.

- On the side of the controllers find the R/L sticker; that is the cover.
- Push the cover downwards towards the wrist strap to remove it.
- Take the old battery out by pushing upwards towards the top of the controller to dislodge where the spring is. This will require a bit of force.
  - You may need to use your nail to help push.

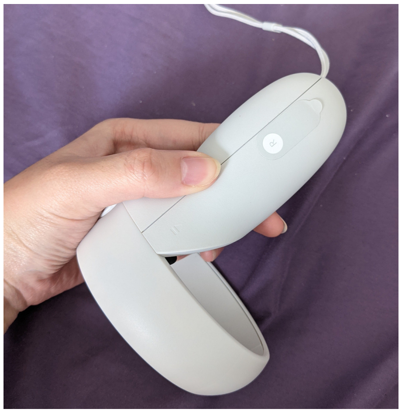

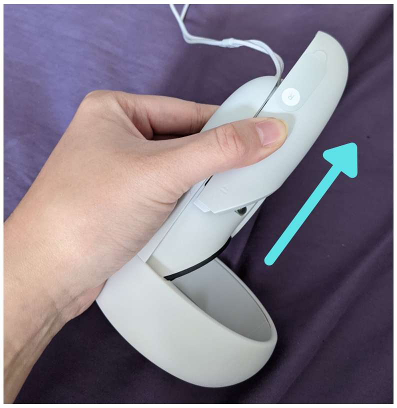

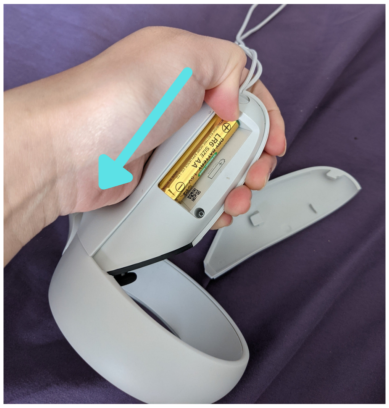

CLEANING THE HEADSET/LENS

We ask that you **clean/wipe** the headset and controllers before and after each session of exercise.

- Wipe down the controllers and the silicon covering in the headset.
  - Use sanitizing wipes and/or spray a cleaner onto a cloth and wipe.
- To clean the lens, ideally use a microfiber cleaning cloth (i.e. glasses cloth).
  - If unavailable, use a soft cloth instead.

Note: If you are using alcohol wipes, please wait up to 5 mins after wiping to use the headset to ensure that it is dry and harsh chemicals will not irritate your skin.

PUTTING AWAY THE HEADSET

After you complete your session please clean the headset, charge it, and put it back into the case.

- Charging cable goes in the pocket.
- Headset is placed facing the pocket, with the strap resting on the small ledge.
  - Make sure the strap is at the appropriate length for it to fit in the case.
- The controllers are placed in the middle within the headset.

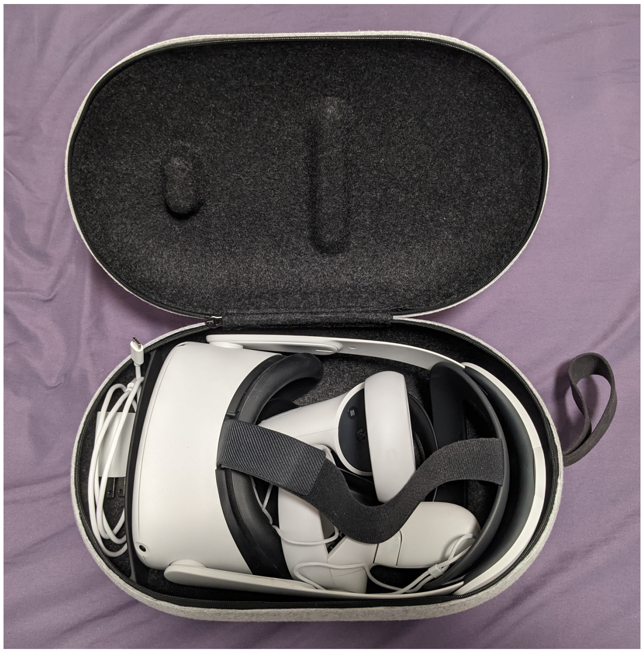

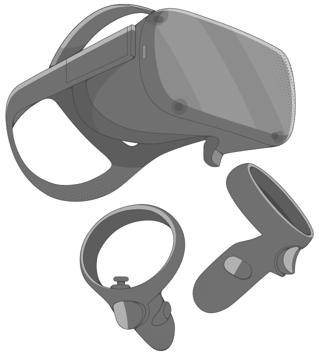

SOFTWARE MANUAL

GETTING READY TO PLAY

1. The VR Exergame is to be completed while **seated only**. Find a spacious area to **sit** (we recommend chairs without arm rests). Make sure there are no objects around you within an arm's reach.
    - Ideally, ask somebody to stay around while you complete the training session.
2. Clean the lenses and silicon covering of the headset
3. Put the eye piece on first before fitting the strap onto the back of your head. Make sure the strap is **extended first** before putting it on your head.
4. Adjust the fit with the back strap by rotating the circle knob at the very back of the headset.
    - **Left** is to **Loosen; Right** is to **Tighten**
5. Adjust the top velcro strap to fit your head shape and help secure the headset.
6. The headset will sense where the controller are. Please have them nearby before putting the headset on. Put the wrist straps on for added safety.
7. Adjust the volume with the volume button located on the bottom right side of the headset

Images seen in the headset should be clear and not blurry. If it is blurry, wipe the lenses and adjust the straps until the headset fits comfortably around your head.

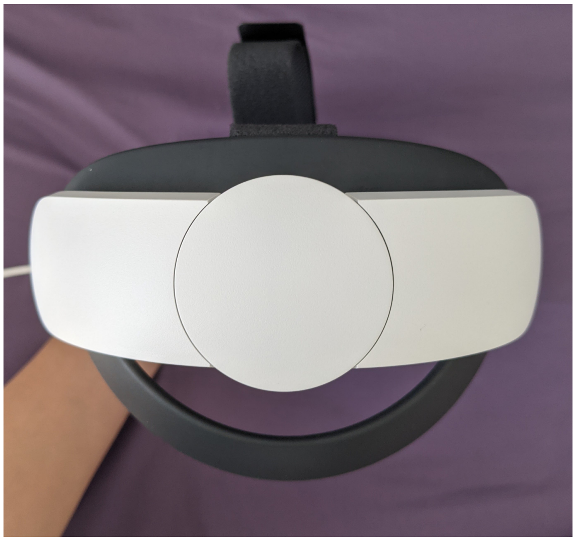
Circle knob to adjust head strap

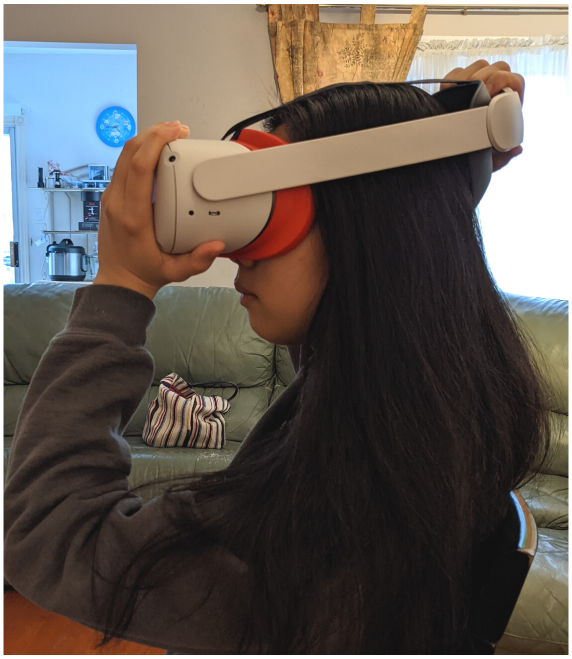
Putting on the headset

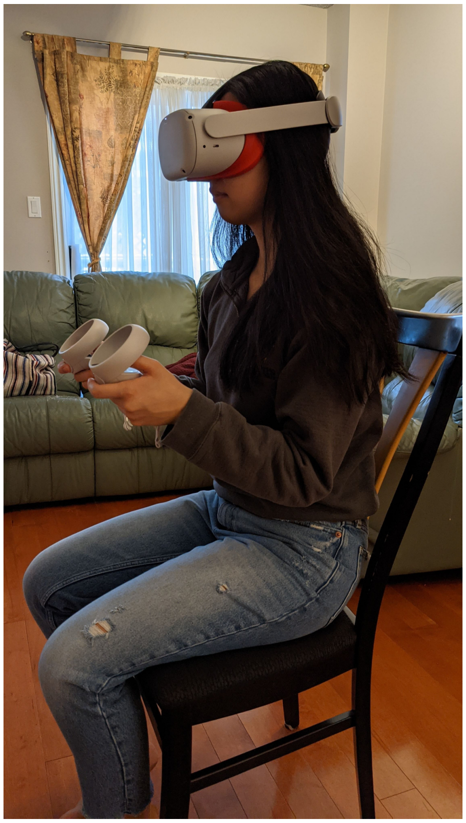
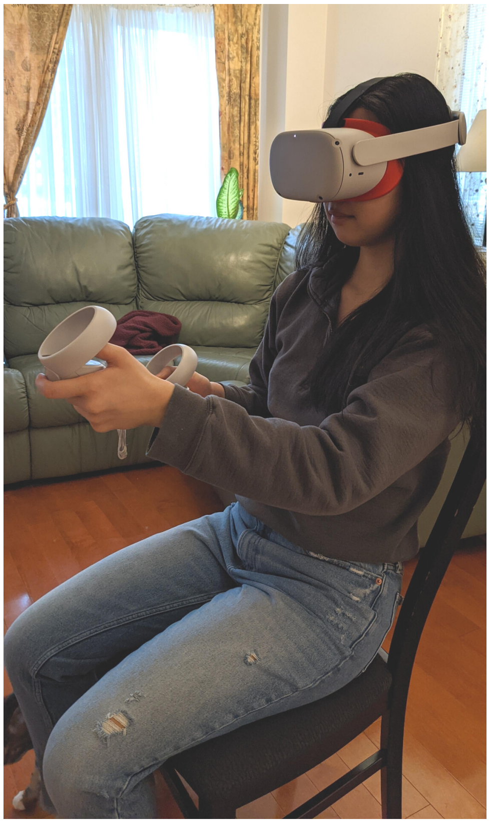
Play the game **seated** on chair with no arm rests and spacious environment

SETTING UP GUARDIAN (CALIBRATION)

To ensure you don’t hurt yourself or others while playing, you must create an imaginary boundary that tells the system where it is safe to play your game. This boundary is called the “Guardian”.
Please set up your Guardian boundary using the following steps:

1. Place your chair in a large space that is clear of things you could knock or hit while you play; ensure everything is a couple of feet beyond arm’s reach.
2. Put on your headset and controllers.
3. To confirm floor level, place the controller onto the ground and select *‘Confirm’*.
4. Choose *‘**switch to stationary boundary**’* and select *"Confirm"*

If you play in a different location than last time, the system should ask you to set up your Guardian boundary again. If you move locations and the system does not ask you to set up a new Guardian boundary, please reset your Guardian boundary using the following steps:

1. Open the Oculus menu with the Oculus button on the right controller.
2. On the very right of the menu select the icon *“Settings”* (⚙)
3. Select *“Guardian”* in the left menu.
4. Select *“Adjust Guardian”* and follow the on-screen instructions to reset.

STARTING UP SEAS THE DAY

After setting up Guardian there will be a large menu that appears, and your background is a lounging area.

1. Access the Oculus Menu by pressing the Oculus button ( ) on the right controller
2. A small Oculus menu will appear just below the large menu. Choose the second icon *"Apps"* (icon with 9 squares)
3. On the top right corner, select the scroll-down menu (it should say *All Apps*) and scroll down using the joystick.
4. Select *"Unknown Sources"*.
5. Seas The Day should appear. Select with the trigger button (index finger) to run the game.

Please note that if the headset does not turn off or restart in between your sessions, you would only need to complete steps 1 and 2.

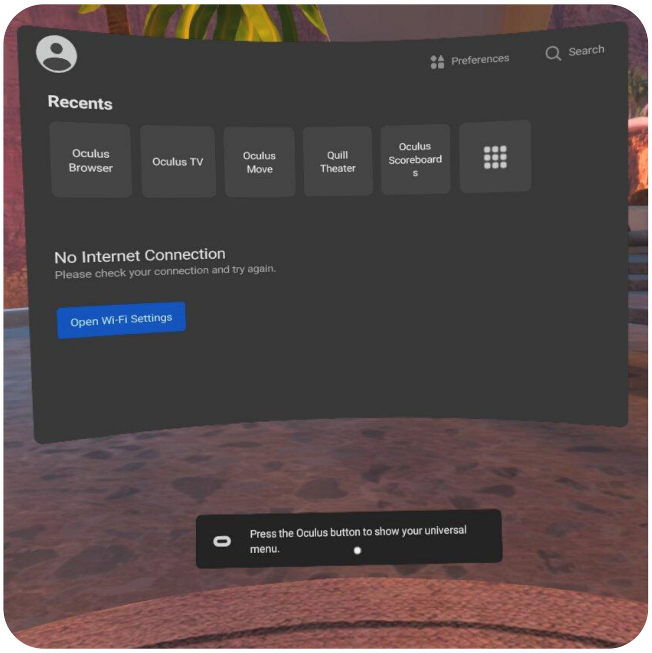

Step 1
Access Oculus menu with Oculus button

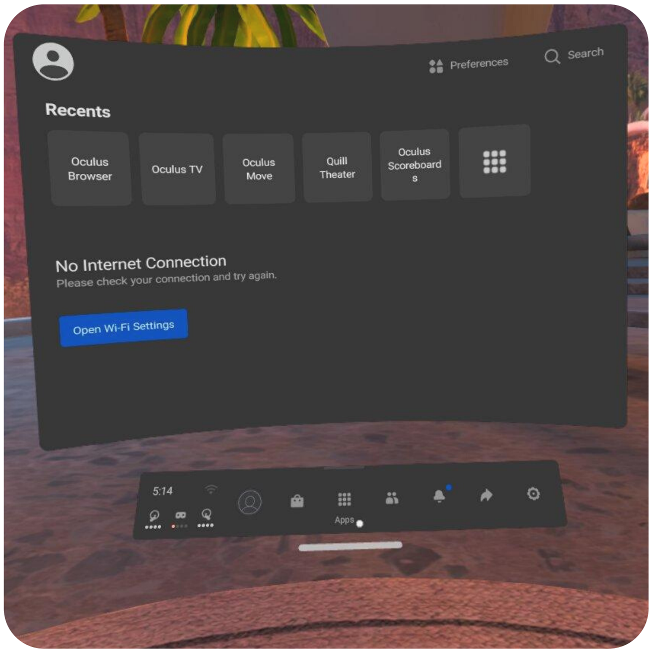

Step 2
Select Apps (2nd icon in the menu)

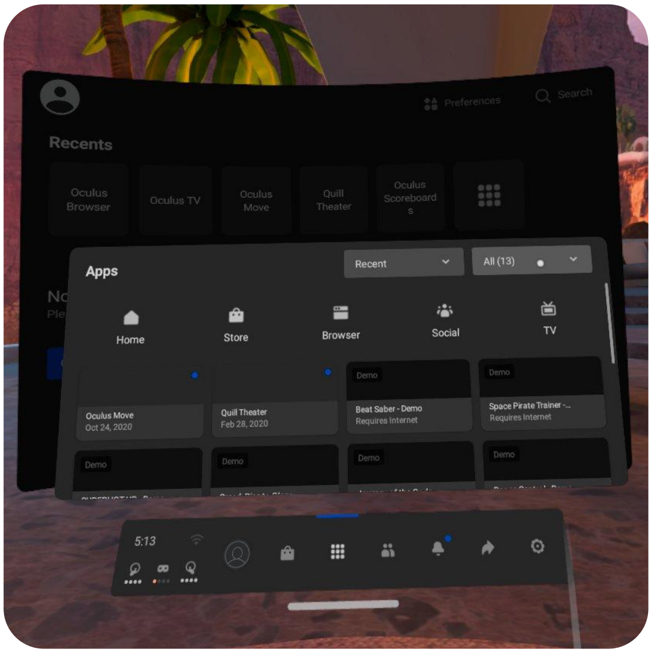

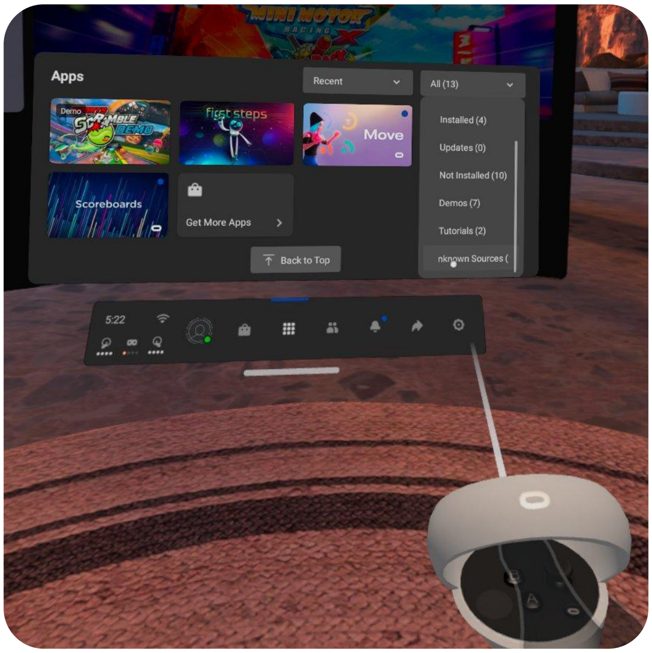

Step 3
Select *All Apps* at the top right corner

Step 4
Scroll down to Unknown Sources

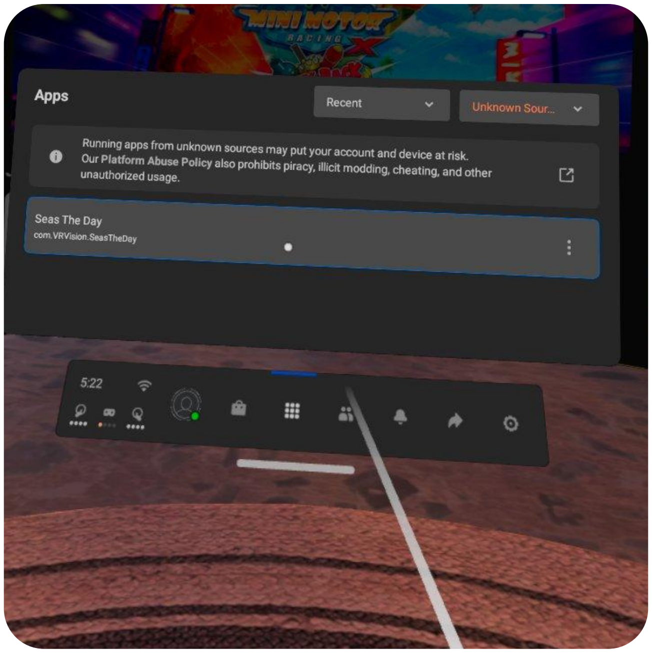

Step 5
Seas The Day should appear

Tai Chi

- Follow the leaf with your hand; it appears slightly below eye level
- Make sure you have the controller in the corresponding hand with the wrist straps on

Rowing

- Make large circular movements with your arms to row and follow the dolphin
- To change to inverted rowing or change duration of the activity follow these steps:
  - In the left controller press the options button (☰) located on the left side. A menu should appear.
  - Select “Settings” and select “Invert Row” and/or change duration

Fishing

- Look for the area where fish are swimming and jumping
- Swing your arm back and swing forward as if you were fishing
- To bring the fish back pull back until the fish comes back to the boat

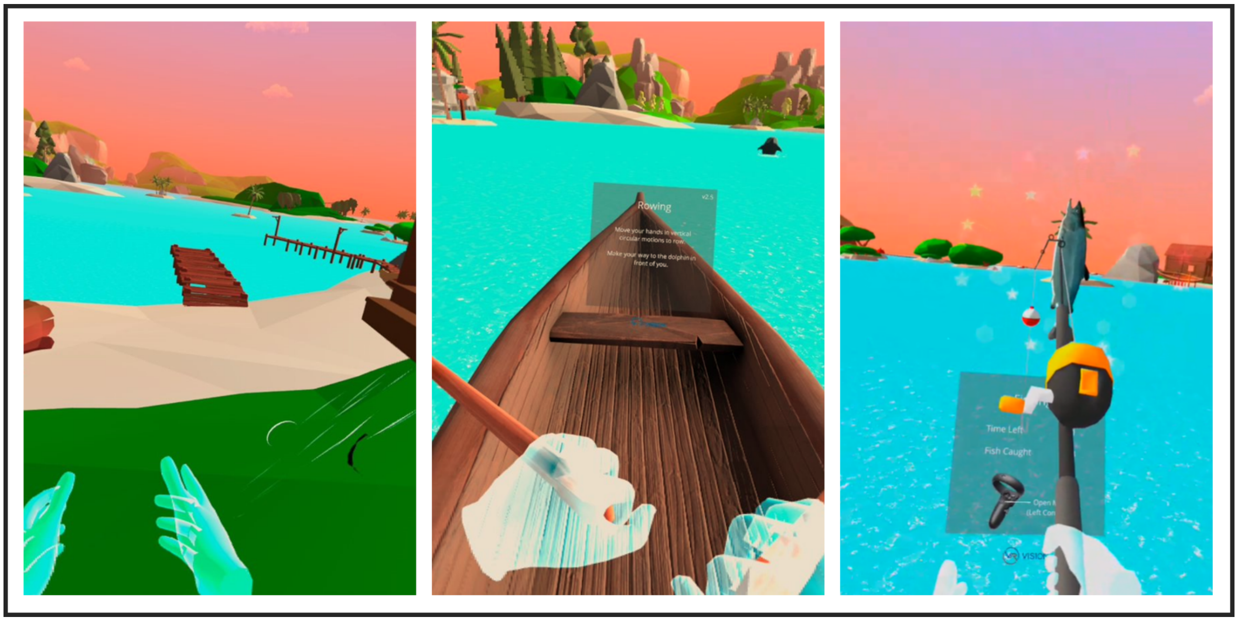

EXITING SEAS THE DAY

After finishing the exergame, you will be prompted to remove your headset. With everything now remote, we ask that you **DO NOT** remove your headset yet and exit the game with these steps:

- Press the oculus button (⬭) on the located right controller, just below the joystick
- The Oculus menu will appear, and choose Quit to return to the main menu

Please **clean and wipe the controllers and silicon covering** on the headset for hygienic purposes.

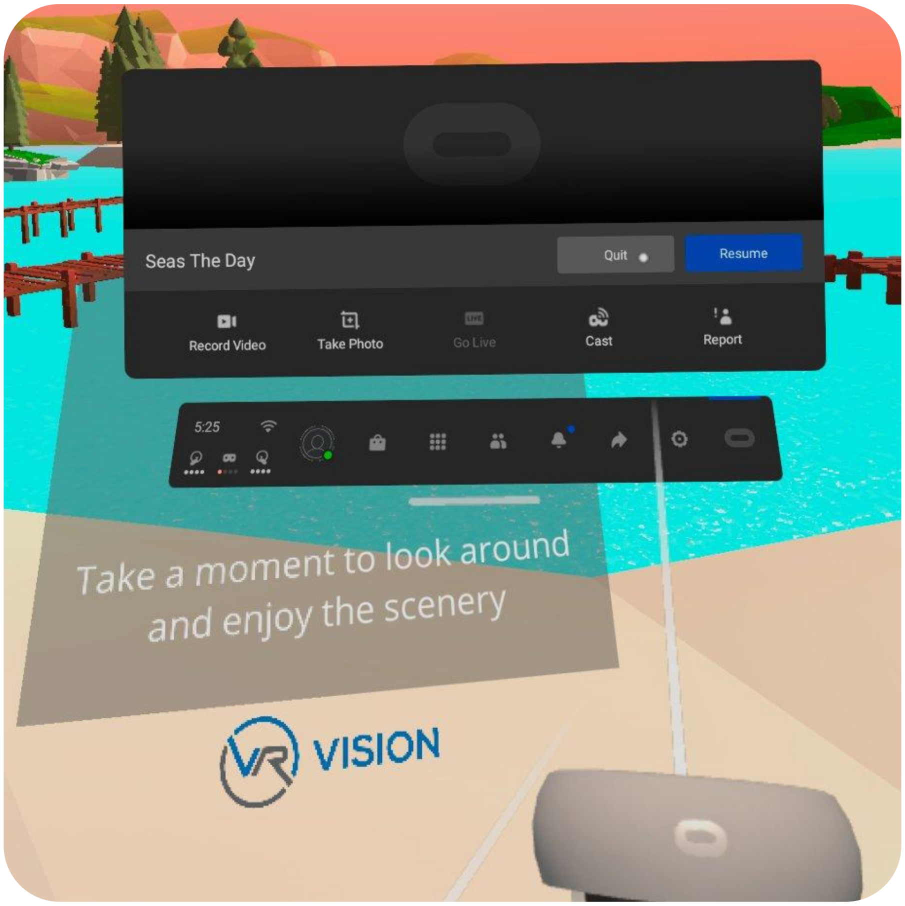

Supplement: Multimedia Appendix 2 [file resprot_v11i6e32955_app2.pdf]
