# Supplementary material for: Immersive Virtual Reality Exergames to Promote the Well-being of Community-Dwelling Older Adults: Protocol for a Mixed Methods Pilot Study
Source: JMIR Res Protoc. 2022 Jun 13;11(6):e32955. doi: 10.2196/32955 (PMC9237784; doi:10.2196/32955)
Supplement: Multimedia Appendix 3 [file resprot_v11i6e32955_app3.pdf]

Immersive virtual reality exergames to promote well-being of community-dwelling older adults: a mixed-methods pilot study protocol

Interview/Focus Group Guide (Experimental Group)

	Topic	Question
**Ice Breaker**		1. What are your prior experiences with games, especially videogames?
**Perceived Usefulness**	**General perception/attitudes towards VR exergaming**	1. Let's talk a bit about your experience playing the VR exergame.<br>a. What are some things you liked about the VR exergames? Why?<br>b. Are there things you did not like about it? What and why?<br>c. What motivated you to play VR Exergames?<br>d. How did your experience differ from your expectations?<br>e. Did you experience challenges when playing VR exergames? If yes, what were the challenges, and how did you overcome them?<br><br>2. Did your enjoyment of the VR exergame change over time? If so, how?<br>a. How was last week different from the first week?

	**Game design elements and appropriateness**<br>**User Experience**<br>**Perceived Enjoyment**	3. How complex/easy were playing VR exergames for you?<br>a. How confident were you playing VR exergame?<br>b. Did you need a lot of support from our research team or a technical person to be able to play VR exergames?<br>c. Did you need to learn a lot of things before you get going with the exergames?<br><br>4. To what extent were you able to follow the instructions and feedback provided by the exergames?<br>a. What could be done to make them better?<br><br>5. How did you incorporate the VR exergames into your daily routines?<br>a. Did you increase your physical activity levels due to the use of the VR exergame?<br>b. Did you replace other physical activities with VR exergames? Which ones?<br>c. Was the use of the VR exergame valuable to supporting physical activity during the COVID-19 pandemic? If yes, why?<br><br>6. How did you feel after playing the VR exergame?<br>a. Did you feel fatigued?<br>b. Did you feel like you were challenged enough?<br>c. How were your mood and alertness?<br><br>7. Were there things about the exergame that made it too difficult? If so, what? What could be done to make this better for you?<br>a. Were there things about the exergame that you felt were not useful or relevant? If so, what and why?<br>b. In what ways (if any) our exergame needs to be modified to be more suitable for you?

Perceived Ease of Use	Technology Adoption Intention to Use	8. Did you feel any pain or discomfort during or after playing the Exergames? If so, please describe.<br>a. Did you feel uncomfortable wearing the headset?<br>b. Did you find any difficulty in using the controllers?<br>c. Did you experience any motion sickness or nausea when playing exergames?<br><br>9. How difficult do you think it would be for you to integrate this technology into your daily routine?<br>a. Would this be different during the COVID-19 pandemic than at other times?<br>b. What could be done to support its use?<br><br>10. How likely is it that you would use VR exergaming in the future?<br>a. Would you recommend exergames to your family/friends?<br>b. What are things you think would keep other older adults from using VR technology for physical activities?<br>c. How might these barriers be overcome?
	Feedback	11. The system was recording some aspects of the gameplay that could be used to give you feedback on your exercise. What would you like to know about your performance and progress?<br><br>12. Is there anything else you would like to add?

Interview/Focus Group Guide (Control Group)

	Topic	Question
**Ice Breaker**		1. What is your previous experience with research? Online research?
**Perceived Usefulness**	**General perception/ attitudes towards online research**	1. Did your enjoyment of participating in this online research study change over time? If so, how?<br>a. Was last week different from the first week? If so, how?
	**Technology Usage**<br>**Online Research Engagement**	1. How did you incorporate technology into your daily life before joining this study?<br>2. Did you increase your technology usage because you are participating in this study?<br>3. Was the use of technology valuable to supporting your social connection during the COVID-19 pandemic? If yes, why?<br>4. Did you feel you needed a lot of support from our research team or someone in your family to be able to complete online tasks? If so, did this change over time?<br>a. Did you find the binders to be helpful?<br>5. How much did you feel you needed to learn to use technology (e.g., connecting to zoom, downloading Firefox, downloading questionnaires, etc.) to participate in this study?

**Perceived Ease of Use**	**Technology Adoption**<br>**Intention to Use**	1. How likely is it that you would participate in an online study in the future?<br>a. Would you recommend it to your family/friends?<br>b. What are things you think would keep other older adults from participating in online research?<br>c. What could be done to support/motivate them?<br>c. How might these barriers be overcome?

	Feedback on Online Assessments	1. How would you describe your experience with online tasks? (cognitive, perceptual, and online questionnaires)<br>a. What was challenging when performing these tasks? (Instructions, required environment, length, complexity, etc.)<br>2. What was your experience with the perceptual and cognitive tasks? These tasks include the flash beep tasks called the RT, SJ, SIFI, and TOJ tasks as well as the task with the arrows called the flanker task.<br>3. Were there things about the online tasks that made it too difficult? If so, what? What could be done to make this better for you?<br>4. How did you feel after each session of cognitive testing (i.e., perceptual and flanker task, TMT, VF)? Did this change throughout the study?<br>a. Did you feel fatigued? If so, when/how often, and what were your symptoms?<br>b. How were your mood and alertness?<br>5. How did you find the instructions (were they clear and easy to follow? What changes would you make to clarify the instructions?)<br>6. What distracted you (if anything) while completing these online tasks?<br>7. In what ways (if any) do our online tasks need to be modified to be more suitable for you?<br>8. Is there anything else you would like to add? Anything regarding this study in general?
